# Genomic features of *Klebsiella* isolates from artisanal ready-to-eat food production facilities

**DOI:** 10.1038/s41598-023-37821-7

**Published:** 2023-07-06

**Authors:** Cecilia Crippa, Frédérique Pasquali, Carla Rodrigues, Alessandra De Cesare, Alex Lucchi, Lucia Gambi, Gerardo Manfreda, Sylvain Brisse, Federica Palma

**Affiliations:** 1 Department of Agricultural and Food Sciences, Alma Mater Studiorum-University of Bologna, Ozzano dell’Emilia, 40064 Bologna, Italy; 2 Biodiversity and Epidemiology of Bacterial Pathogens, Institut Pasteur, Université Paris Cité, 75015 Paris, France; 3 Department of Veterinary Medical Sciences, Alma Mater Studiorum-University of Bologna, Ozzano Dell’Emilia, 40064 Bologna, Italy; 4 Biological Resource Center of Institut Pasteur, Institut Pasteur, Université Paris Cité, 75015 Paris, France

**Keywords:** Bacteriology, Bacterial genomics

## Abstract

Increasing reports on *K. pneumoniae* strains with antimicrobial resistance and virulence traits from food and farm animals are raising concerns about the potential role of *Klebsiella* spp. as a foodborne pathogen. This study aimed to report and characterize *Klebsiella* spp. isolates from two artisanal ready-to-eat food (soft cheese and salami) producing facilities, and to track similar genotypes in different ecological niches. Over 1170 samples were collected during the whole production chain of different food batches. The overall *Klebsiella* prevalence was 6%. Strains were classified into the three *Klebsiella* species complexes: *K. pneumoniae* (KpSC, n = 17), *K. oxytoca* (KoSC, n = 38) and *K. planticola* (KplaSC, n = 18). Despite high genetic diversity we found in terms of known and new sequence types (STs), core genome phylogeny revealed clonal strains persisting in the same processing setting for over 14 months, isolated from the environment, raw materials and end-products. Strains showed a natural antimicrobial resistance phenotype-genotype. *K. pneumoniae* strains showed the highest virulence potential, with sequence types ST4242 and ST107 strains carrying yersiniabactin *ybt16* and aerobactin *iuc3*. The latter was detected in all *K. pneumoniae* from salami and was located on a large conjugative plasmid highly similar (97% identity) to *iuc*3^+^ plasmids from human and pig strains circulating in nearby regions of Italy. While identical genotypes may persist along the whole food production process, different genotypes from distinct sources in the same facility shared an *iuc*3-plasmid. Surveillance in the food chain will be crucial to obtain a more comprehensive picture of the circulation of *Klebsiella* strains with pathogenic potential.

## Introduction

The *Klebsiella* genus belongs to the *Enterobacteriaceae* family and comprises capsulated Gram-negative, rod-shaped, facultative anaerobic bacteria, which are ubiquitously found in a wide range of host-associated and environmental niches, including soil, surface waters, plants and gastrointestinal tracts of animals and humans^1,2^. *Klebsiella* encompasses several species including pathogenic strains of important public health concern in nosocomial settings (e.g., *K. pneumoniae*). The *Klebsiella pneumoniae* species complex (KpSC) currently includes seven major phylogroups: *K. pneumoniae* sensu stricto (Kp1), *K. quasipneumoniae* subsp. *quasipneumoniae* (Kp2), *K. variicola* subsp. *variicola* (Kp3), *K. quasipneumoniae* subsp. *similipneumoniae* (Kp4), *K. variicola* subsp. *tropica* (Kp5), *K. quasivariicola* (Kp6) and *K. africana* (Kp7)^3–8^. Similarly, the *Klebsiella oxytoca* species complex (KoSC) comprises nine phylogroups, from Ko1 to Ko9, defined in accordance to the chromosomal variants belonging to the classe A beta-lactamase (*bla*_OXY_) gene, with *K. michiganensis* (Ko1) including the Ko5 sublineage^9^, *K. oxytoca* sensu stricto (Ko2), *K. spallanzanii* (Ko3) embracing the sublineage Ko9^10^, *K. pasteurii* (Ko4), *K. grimontii* (Ko6), *K. huaxensis* (Ko8) and the undetermined taxonomic status of Ko7^11,12^. *Klebsiella planticola* was firstly described in 1981^13^, whereas *K. ornithinolytica* was introduced 8 years later^14^. In 2001, based on the molecular analysis of 16S ribosomal RNA (rRNA) and RNA polymerase β subunit encoding genes (*rpoB*), *K. planticola* and *K. ornithinolytica* were classified into a new genus, named as *Raoultella*^15^. Thirteen years later, a new species *Raoultella electrica* was discovered^16^. A reunification of *Raoultella* and *Klebsiella* into the single genus *Klebsiella* has subsequently been proposed, since phylogenomic analyses showed that the genus *Raoultella* is nested within *Klebsiella*, thus not supporting the assignment of *Raoultella* species as a separate genus. According to this reclassification, the *Klebsiella planticola* species complex (KplaSC) encompasses four phylogroups: *K. planticola* (Kplan1), *K. ornithinolytica* (Kplan2), *K. electrica* (Kplan3) and Kplan4 representing an undescribed species^17^.

*K. pneumoniae* and to a lesser degree *K. oxytoca,* are associated to hospital-acquired and community-onset infections such as bacteremia, pneumonia, meningitis, and urinary tract infections, which are particularly worrisome in immunocompromised individuals^18–20^. One of the major concerns with *Klebsiella* infections, especially with *K. pneumoniae*, is the emergence and dissemination of isolates producing extended-spectrum β-lactamases (ESBL) and/or carbapenemases, and showing multi-drug resistance (MDR), which impairs the clinical management of healthcare-associated infections^21^. This has led to the declaration of *K. pneumoniae* as a critical priority pathogen by the World Health Organization (WHO)^22^. Besides nosocomial bacteremia, invasive community-acquired infections are mostly linked to so-called hypervirulent *K. pneumoniae* strains (hvKp). Initially described in Asian countries, hvKp isolates commonly harbor horizontally-acquired virulence factors encoding for siderophores systems such as yersiniabactin (Ybt), aerobactin (Iuc/iut) and salmochelin (Iro)^23^.

Beyond clinical settings, *Klebsiella* spp. have been also found in several food products. Whilst not typically considered as foodborne pathogens, *Klebsiella* spp. isolates in food deserve further investigation for several reasons^24^. First, *Klebsiella* spp. ability in colonizing the gastrointestinal tract after food consumption often precedes infection^25,26^. Second, antimicrobial-resistance (AMR) or virulence genes, mostly present in mobile genetic elements, could be transferred to other pathogens found in the same ecosystem, leading to the emergence of new resistance or pathogenesis mechanisms. In this regards, some studies pointed out that *K. pneumoniae* could potentially act as reservoir of AMR genes in the food chain, colonizing poultry and meat products^27–29^, and/or of genetic elements associated with enhanced virulence (K1, K2, and K54 capsular serotypes and *wcaG*) in raw or ready-to-eat (RTE) foods^24^.

At present, knowledges on the prevalence rates of antibiotic resistant and/or virulent *Klebsiella* spp. isolates from food chains is limited. In particular, artisanal productions are widely appreciated among consumers in Italy, and contribute to the national cultural heritage. While for some products the authenticity of traditional production methods is protected by European legal designations (e.g., PDO, PGI and STG), others result in lack of production standardization. Non-standardized productive processes combined with missing extensive automation in small-scale facilities could pose severe hazards in terms of final product’s microbial safety, especially when products result from short fermentations and/or are consumed without cooking. Examples of safety issues associated to artisanal fermentative processes are represented by biological (foodborne pathogens) or chemical (mycotoxins or biogenic amines) contaminants generated in the context of spontaneous fermentations, which could represent a severe hazard for human health^30^.

Given the above, we aimed to investigate *Klebsiella* spp. isolated from the artisanal food chain to gather evidence on the public health risk that artisanal RTE foods may pose as vehicles of *Klebsiella* strains. We focused our study on *Klebsiella* strains (broadly comprising its different species complexes *K. pneumoniae*, *K. oxytoca* and *K. planticola*) repeatedly isolated from two Italian artisanal RTE food productions (salami and cheese) over a sampling period covering six commercial batches. We assessed the occurrence of *Klebsiella* spp. strains through the selected food productions and performed genomic sequencing to improve their taxonomic characterization and describe the circulating genotypes and their genetic features of proven clinical importance (i.e., antimicrobial resistance and virulence). Finally, we compared our sequences with public databases to evaluate the genetic relatedness between our food isolates and previous ones from other ecological niches.

## Results

### *Klebsiella* spp. prevalence and taxonomic assignment across food processing facilities

**Figure 1:**
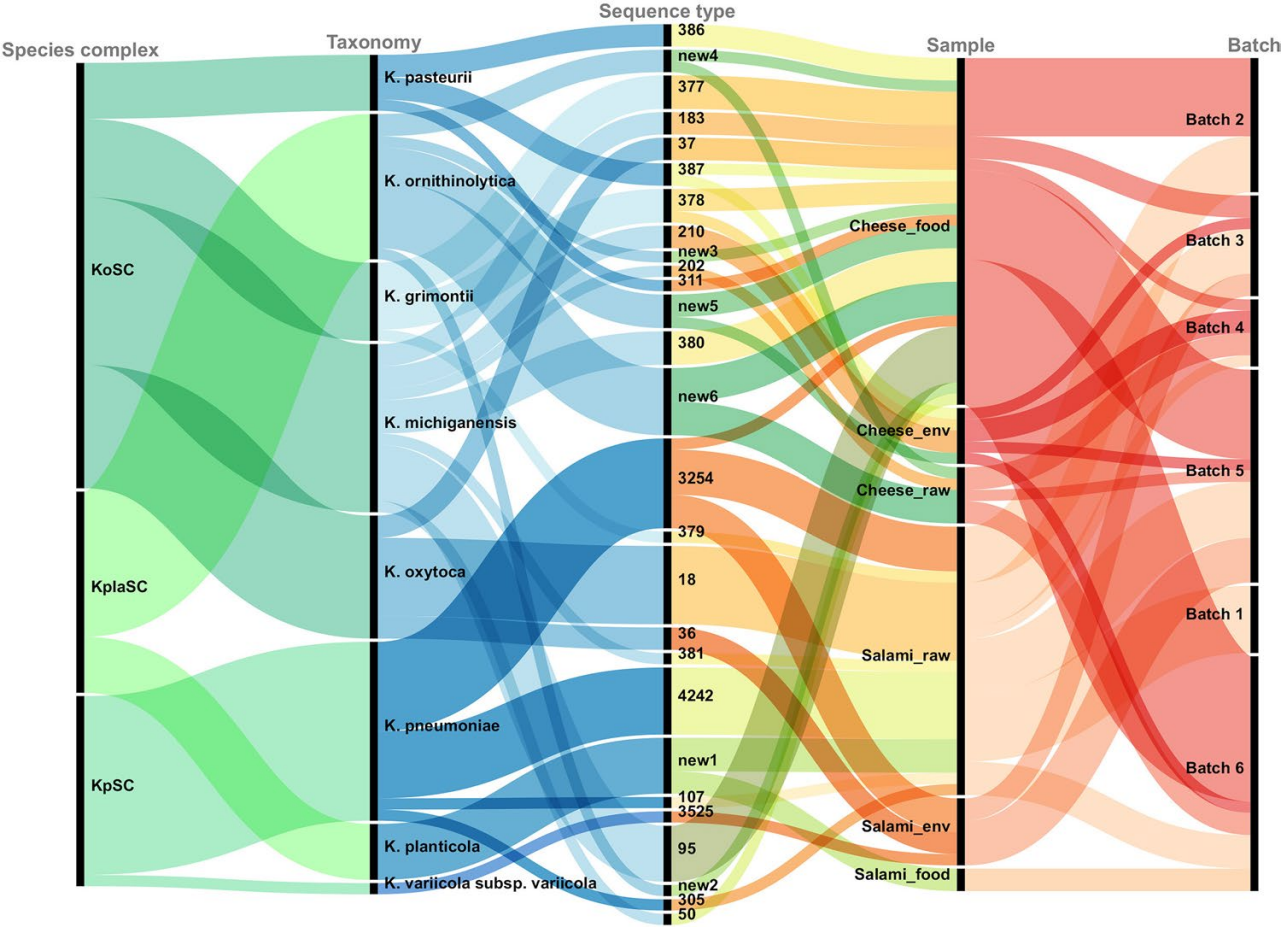
Distribution of Ko, Kpla and Kp species complexes and related sequence types in cheese and salami artisanal facilities (the environment: env; intermediate: raw and final products: food), batches as well as sequence types. The diagram was created with RAWGraphs 2.0 beta (https://app.rawgraphs.io/).

Over 1170 samples were collected across six commercial batches sequentially produced from January 2020 to May 2021 from two artisanal food production chains (cheese and salami). The overall prevalence of the *Klebsiella* spp. strains across both productions was 6%. In total, 75 *Klebsiella* isolates were found and identified as *K. oxytoca* (n = 52, 69%) and *K. pneumoniae* (n = 23, 31%) based on biochemical test (RapID™ ONE System, Thermo Scientific™) and multiplex-PCR^31^ (Supplementary Table S1), followed by whole-genome sequencing (WGS). Using genome-based taxonomic designations, 73 out of 75 isolates were classified in 3 *Klebsiella* species complexes: 17 (23%) KpSC isolates, including 16 *K. pneumoniae* sensu stricto and 1 *K. variicola* subsp. *variicola*; 38 (52%) KoSC of which 11 *K. oxytoca* sensu stricto, 15 *K. michiganensis*, 5 *K. pasteurii* and 7 *K. grimontii*; and 18 (25%) KplaSC with 13 *K. ornithinolytica* and 5 *K. planticola* (Supplementary Table S1, Fig. 1). Two isolates previously identified as *Klebsiella* spp. were re-classified as *Citrobacter* spp. and thus excluded from the dataset. In summary, using genomics data, we were able to improve species identification for the 28% of the isolates previously identified by biochemical and molecular testing (Supplementary Table S1).

*Klebsiella* spp. isolates were detected in all batches of salami and five of six batches of cheese, and were isolated along approximately the whole sampling period (Fig. 1, Supplementary Table S1). The species distribution across food productions, batches and sample origin showed a higher recovering of KpSC isolates from salami (94%; n = 16/17) and KoSC isolates from cheese (71%; n = 27/38). Regarding KplaSC, *K. ornithinolytica* isolates were retrieved from four batches of cheese samples (collected in warm and maturation room as well as stored cheese) and in the associated environment. On the other hand, *K. planticola* detection was restricted to the salami production and represented strains collected from the final product (RTE salami with 28-weeks ripening). Strain’s taxonomy assignments, distribution across samples and batches are showed in Fig. 1.

Draft genomes generated through de novo assembly of Illumina short reads showed good contiguity metrics (Supplementary Fig. S1): low number of contigs (33–280 bp) and high N50 (161,990–1,140,929); genome length (5.3–6.5 Mb), GC contents (54.9–57.4%) and number of predicted coding sequences (CDS: 4900–6193), in the typical range for *Klebsiella* spp. (Supplementary Table S2). The median values of number of contigs, cumulative size, and number of CDSs were higher for the KoSC (85 contigs, 6.08 Mb and 5,640 CDSs) and KplaSC (72 contigs, 5.8 Mb and 5,404 CDSs) compared to KpSC (43 contigs, 5.4 Mb and 5,008 CDSs). On the other side, KpSC presented the highest GC content (~ 57%) in comparison to ~ 55% of other *Klebsiella* species complexes.

### Antimicrobial susceptibility testing

Results from antibiotic susceptibility testing (AST) were obtained for 72 out of 75 isolates (Supplementary Table S3) and 71 showed a natural antibiotic susceptibility phenotype. All isolates except one showed resistance to ampicillin (MIC ≥ 32), as expected for *Klebsiella* spp*.* due to the presence of an intrinsic chromosomal type A beta-lactamase. The exception was one *K. ornithinolytica* strain collected from cheese with a MIC = 8, which is the upper limit to be considered as susceptible^32^. Only two isolates (from cheese and salami productions) showed resistance to azithromycin (MIC = 32).

### Sequence types distribution

We recovered sequence types (STs) for all isolates except KplaSC ones, based on the 7-MLST loci schemes available in BIGSdb-Pasteur for KpSC (https://bigsdb.pasteur.fr/klebsiella/) and PubMLST for KoSC (https://pubmlst.org/organisms/klebsiella-oxytoca) (Supplementary Table S4). Overall, 21 STs were identified for KpSC and KoSC, including 9 novel STs. We identified 4 known and 1 new ST (defined as ST5929) within the KpSC, as well as 8 known and 8 new STs (defined as ST311, ST377, ST378, ST379, ST380, ST381, ST386, ST387) within the KoSC (Supplementary Table S4). The 35% and 41% of KpSC isolates were respectively designed as ST4242 and ST3254. ST3254 was found in two salami batches in raw materials, ripened salami (up to ten weeks) and the environment, suggesting that this strain likely persisted for several months in the processing environment. In contrast, ST4242 was only found in raw material samples from salami but distributed among different batches, suggesting a possible introduction in the food production line from animals raised in livestock farming. A similar distribution was reported for KoSC strains, with isolates sharing the same unique ST even though they came from the different sample’s origins (environment and stored cheese) or batches (Fig. 1).

### Phylogenetic analyses and population structure

**Figure 2:**
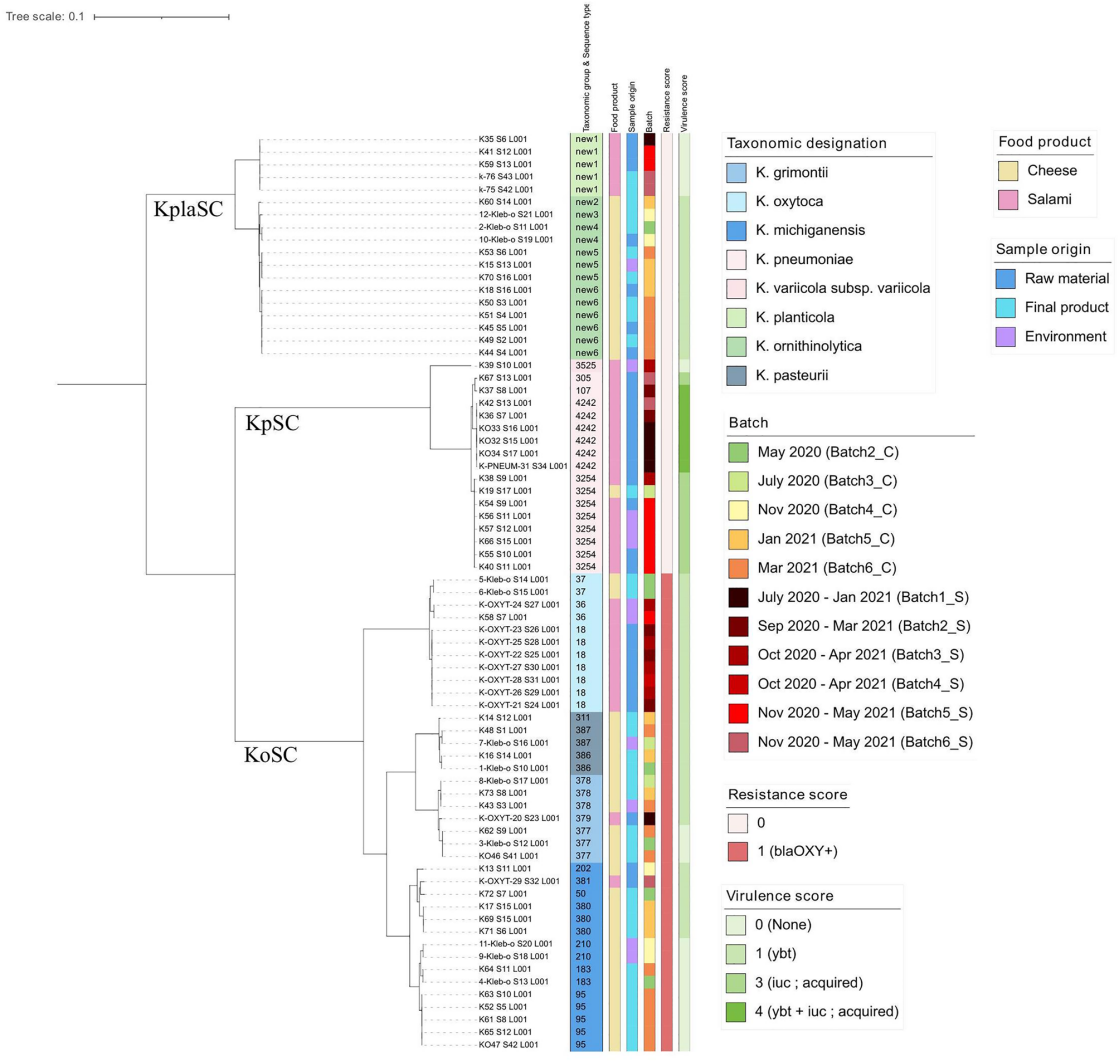
Maximum likelihood phylogenetic tree inferred from core gene alignments of 73 *Klebsiella* genomes isolated from two Italian meat and dairy artisanal food productions. Species complexes (Kpla, Kp and Ko) are indicated along the branches. The tree was rooted with the KplaSC. Three main clusters correspond to the species complexes with color highlights in the first columns indicating the ST: KplaSC (green), KpSC (pink) and KoSC (blue). Taxonomic designation as well as sequence type, if available, are indicated. Sample metadata (food product, sample origin and batch) and resistance and virulence scores given by Kleborate are shown on the figure color key on the right.

To address the question of strains relatedness and genotypes circulation across artisanal facilities, we inferred a maximum likelihood (ML) phylogenetic tree from the core-genome alignment. As expected, the phylogenomic reconstruction shaped three distinct major clades according to the *Klebsiella* species complexes (Fig. 2). Within each clade, strains clusters and subclusters were displayed according to the species and, more in depth, to the ST, with strains from different STs but harboring similar virulence features grouped into tight branches. For each species complex, clonal groups of isolates (i.e., clonal genotypes—corresponding to the same ST in KpSC and KoSC) were contaminating the processing facilities overtime. Clonal genotypes were detected across different origins (raw materials, food and environment) and all along the whole production of batches, from raw materials to the final products (i.e., the same *K. planticola* genotype was found from minced meat up to 28 weeks ripening salami).

A cgMLST approach was used to investigate clonal relationships of our *Klebsiella* spp. from artisanal food in the context of selected public genomes (n = 94) representing the same taxonomic species and STs. We identified < 1.5% of pairwise allele distances (8–9 different alleles) among ST3254 strains sampled from salami (n = 8) and human carriage from Africa (n = 2), using the 629 loci cgMLST scheme hosted in the BIGSdb *Klebsiella* database^33^ (Supplementary Table S5). We further identified a ~ 1.5% genetic distance (50 allele variants across a 3272-loci cgMLST scheme designed in this study) in a cluster of ST37 *K. oxytoca* isolates from the Italian cheese sampled along its shelf-life (n = 2) and human carriage and environmental *K. oxytoca* strains isolated in Europe (n = 6). No clusters were observed at ≤ 1.5% pairwise distance for KplaSC isolates from this study and public repositories using the here designed 2957-loci cgMLST scheme (Supplementary Table S6).

### Detection, distribution and mobilization of AMR, virulence, and serotype determinant genes

Virulence and AMR genes detected by Kleborate were summarized in Supplementary Table S7. Overall, few antimicrobial resistance determinants were found as expected from the AST results. KpSC strains carried only the intrinsic β-lactamase-encoding genes *bla*SHV (*K. pneumoniae*) and *bla*LEN (*K. variicola*). KoSC strains from both artisanal productions harbored the β-lactamase-encoding *bla*_OXY-1_, *bla*_OXY-2_, *bla*_OXY-4_, *bla*_OXY-5_ and *bla*_OXY-6_ resistance genes, which lifted their resistance score to 1, although these genes are described as typically intrinsic among this SC. Few other AMR genes were detected, such as to streptomycin (*str*AB) in two *K. pasteurii*, with one of them also harboring the sulphonamide resistance gene *sul*2.

On the other hand, several virulence features and strains with high virulence score were found. Although no virulence loci were found in *K. planticola* isolates (score 0), yersiniabactin was detected in the 68% (n = 26) of KoSC isolates and in all *K. ornithinolytica* genomes (consistent with a previous report^34^), which were classified as virulence score 1. The highest scores were assigned to *K. pneumoniae* sensu stricto isolates, with 9 isolates of virulence score 3 carrying the acquired *iuc3* (aerobactin sequence type AbST43) and 7 isolates of virulence score 4 harboring *iuc3* (with a novel combination of *iuc* alleles defined as AbST96) in combination with the yersiniabactin *ybt16* locus. The yersiniabactin profile presented a novel allele variant which was curated in BIGSdb-Pasteur and defined as YbST600. Virulence score 3 comprised all isolates of the ST3254 clone and the ST305 isolate, while all isolates of the ST4242 clone and the ST107 isolate showed virulence score 4.

**Figure 3:**
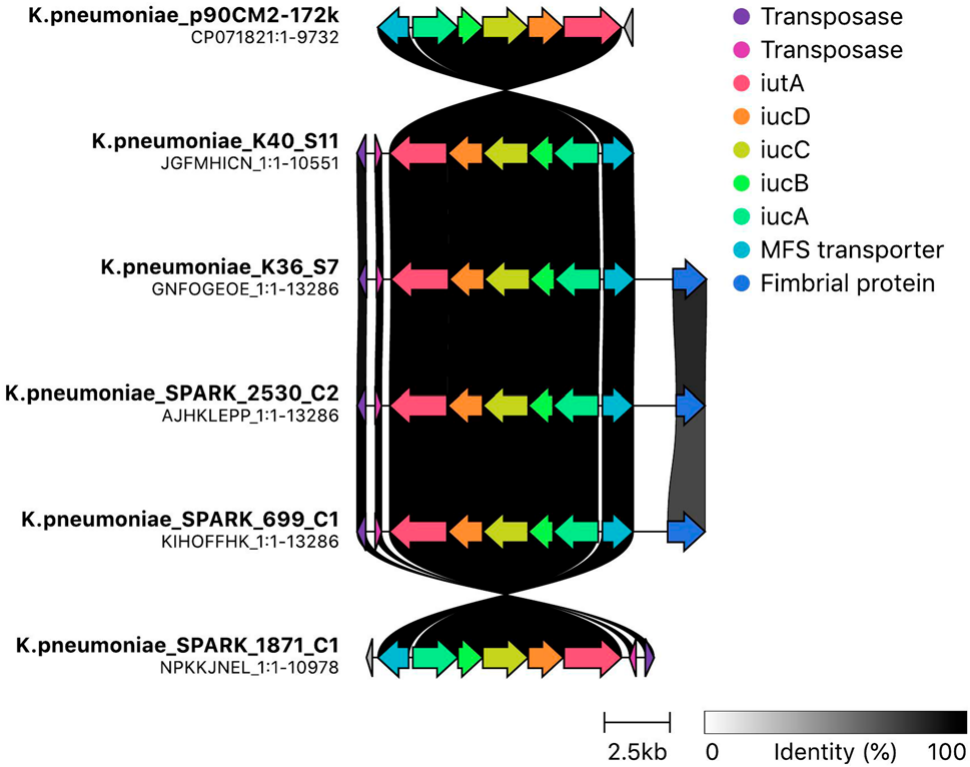
Comparison of the plasmid-encoded *iuc*3 locus between *K. pneumoniae* from food, pig farming and clinical settings. Clinker visualization shows gene cluster comparison of the plasmid-encoded aerobactin locus *iuc*3 (*iucABCD, iutA*) and other flanking genes encoding for transposable elements (transposase), membrane transporters (Major Facilitator Superfamily (MFS) transporters) as well as fimbrial proteins. Gene clusters belonged to close circular plasmid reference (K.pneumoniae_p90CM2-172k) from a public strain, salami (K.pneumoniae_K40_S11; K.pneumoniae_K36_S7), pig farming (K.pneumoniae_SPARK_2530_C2; K.pneumoniae_SPARK_1871_C1) and clinical settings (K.pneumoniae_SPARK_699_C1).

Notably, *ybt16* was located on the integrative conjugative element ICEKp12, while *iuc3* was observed on a conjugative plasmid sequence with IncFIB/IncFII replicon type, classified as pMLST FIIK_2 in ST4242 isolates and FIIK_10 in ST3254 and ST107 isolates (Supplementary Table S8). Given the critical role of aerobactin in virulence and invasive disease, we compared the *iuc3*^+^ plasmid sequences of our *K. pneumoniae* isolates with three *iuc3*^+^ plasmid types found in *K. pneumoniae* isolated from pig livestock and hospitalized patients in the nearby area of Pavia city (Northern Italy) between 2018 and 2017^34^. We found a very high similarity (ANI > 98%) among all sequences (Supplementary Table S9) and similar pMLST profiles. Gene cluster comparison (https://github.com/gamcil/clinker) of one annotated sequence for each replicon type/pMLST combination (Supplementary Table S10) and a close circular plasmid p90CM2-172k (ANI 97%; ~ 70 × coverage) from a public strain (accession number NZ_CP071821) showed a high conservation of the *iuc*3 locus (*iucABCD*/*iutA*) in strains from our food facilities with those from a clinical case (699_C1) and a pig (2530_C2 and 1871_C1) collected in the same geographical area (Fig. 3).

### Pan-genome repertoire

Pan-genome analysis performed on the 73 *Klebsiella* spp. genomes clustered all protein-coding sequences into 19,365 groups of orthologues (Supplementary Fig. S2). Of these, only 15% (n = 2911) were conserved across isolates from different species. By contrast, 85% (n = 16,454) of genes represented the accessory genome and were distributed among shell (n = 5802) and cloud (n = 10,652) genes, respectively found in 15–95% and less than 15% of genomes. The cloud genes accounted for the 55% of all genes detected across the full set of genomes. Despite the heterogeneity of the gene repertoire, we observed several clusters of isolates harboring the same groups of orthologues (Supplementary Fig. S2).

## Discussion

AMR is a global public health threat strongly heightened by the dissemination of clinically relevant antimicrobial resistance genes and pathogens between clinical, community, agricultural and environmental settings^35^. The food-producing environment represent a possible reservoir of antimicrobial-resistant bacteria which can spread throughout the food chain, posing a potential hazard to public health^36^. *Klebsiella pneumoniae* is one of the priority pathogens members of the ESKAPE group (*Enterococcus faecium*, *Staphylococcus aureus*, *K. pneumoniae*, *Acinetobacter baumannii*, *Pseudomonas aeruginosa*, *Enterobacter* spp.)^37^. It represents a leading cause of drug-resistant hospital-acquired or nosocomial life-threatening infections and has been prioritized by the WHO as a global critical pathogen contributing to the greatest AMR burden^22^. The ability of *Klebsiella* species to act as a key trafficker of AMR genes following its wide ecological spread, the high AMR gene diversity and plasmid load^25^ combined with the increasing reports suggests a foodborne transmission capacity^24,28,38,39^, emphasizing the importance of monitoring the spread of such species through the food chain. In the present study, we investigated the microbiological hazards of Italian RTE food artisanal production systems, an unexplored environment so far, to study their potential role as a source of *Klebsiella* spp. strains with pathogenic potential. We focused our investigation on cow milk-based soft cheese and pork meat-based salami because these are among the most consumed RTE food subcategories in the EU and are often associated with foodborne diseases^40,41^.

Across the 1,170 samples collected, we observed a low prevalence of *Klebsiella* spp. (6%), with 73 strains identified among the two facilities considered together. Identification results from conventional and genomic methods agreed for only 72% of strains, confirming that biochemical and multiplex-PCR tests, commonly applied in food laboratories, are not as reliable as WGS, especially to distinguish close relatives within *Klebsiella* species complexes. While *K. ornithinolytica* and *K. planticola* strains were rare and associated with a specific production (cheese and salami respectively), KoSC and KpSC strains occurred in both production systems. However, 94% of KpSC strains were observed in the salami production, whereas 71% of KoSC were found in cheese production. *K. oxytoca*, *K. pneumoniae*, *K. michiganensis* or *K. ornithinolytica* strains have been already found worldwide in dairy and meat products^42–46^, and their associated raw materials and production environments^47^. *Klebsiella* spp. were also isolated from minced pork meat fermentation processes in Belgium^48^ and in Spanish raw pork sausages before the ripening process^49^. Our observations corroborate such findings and suggest that *Klebsiella* spp. clones can colonize dairy and meat food productions over several months. Such strains may come from the animal slaughtering, and/or other processes at farm level, or persist in the facilities through contaminated environment or operators. Here, we provide the first evidence on the presence of several *Klebsiella* spp. populations in two Italian artisanal productions of ready-to-eat foods. Despite the high heterogeneity and genetic diversity at the strain level, which is typical for such populations^25,50^, we found that several clones were contaminating both processing environments and food products. Some of the detected clonal genotypes (e.g., new6 of *K. ornithinolytica*) lasted for several weeks or months along the whole production (from raw material to the final product), showing the capacity of *Klebsiella* to persist in this environment. Whether strains were repeatedly introduced in the artisanal food processing from the livestock or transmitted along the production by the food operators or persisting in the facilities due to cleaning and disinfection failures, will need to be addressed in future studies.

Although data from the food sector are still limited, events of genes’ acquisition/loss in *Klebsiella* spp. population through horizontal gene transfer (HGT) are frequent during niche adaptation^8^. In a clinical context, the presence of mobile genetic elements (MGE) carrying virulence and antibiotic and metal resistance genes confers an advantage during host colonization or infection^51^. Indeed, chromosomal recombination processes and acquisition of various AMR genes on diverse plasmids can shape the evolution of MDR clonal groups, such as CG258^52^. The large repertoire of accessory genes (encompassing several encoding for virulence functions linked to invasive disease), that characterizes animal and human KpSC strains collected worldwide, emphasizes the considerable genomic plasticity of such species^50^. This also emphasizes the need of gathering additional data on the distribution of concerning genes in strains from other important sources, such as food, to better understand horizontal gene transfer dynamics and *Klebsiella* spp. niche adaptation.

Our study showed that the virulence locus *iuc*3 was located on a large conjugative plasmid that has been likely transferred within and between genotypes contaminating the Italian facilities.

Investigating the genomic context of *iuc3* raised the possibility of a hypothetical transmission of virulence traits between isolates collected from different settings (food, clinical, environmental), while providing insights on the potential public health risk of *Klebsiella* isolated from artisanal food. The fact that *iuc3*-harbouring plasmids from the artisanal food chain were similar (> 97%) to plasmid sequences of *K. pneumoniae* strains isolated from human and pig livestock in close geographical areas, may suggest a possible transfer of virulence plasmids across such sectors. A recent genome-based analysis performed on animals and food products in Germany^2^ proposed domestic pigs as a reservoir for *K. pneumoniae* plasmids carrying *iuc*3. Likewise, high occurrence of the aerobactin lineage *iuc*3 located on potentially conjugative IncFIBK/FIIK plasmids in *K. pneumoniae* has been recently described from healthy fattening pig populations in Norway^53^. A lower frequency was also reported in pigs from Thailand^54^ and Guadeloupe^55^ (French West Indies) as well as from wild boars in Central Italy^56^, suggesting a possible spread and adaptation of this siderophore lineage to the porcine reservoir over broad geographical territories.

Evidence provided by our results on the circulation of *iuc*3^+^ *K. pneumoniae* clones in the raw pork meat and its processing environment and their similarity with *iuc3*-harbouring plasmids from nearby pig livestock, contributed to add new exclusive insights on aerobactin *iuc*3 lineage circulation across the food industry and propounds the view that pigs may act as an important reservoir. However, addressing if pigs represent the originating source of *iuc*3^+^ *K. pneumoniae* from our study is a key aspect which deserves to be uncovered in further studies by extending the sampling to the whole production cycle from animals farming and/or slaughtering.

A more concise understanding of the source of *iuc*3^+^ *K. pneumoniae* is also required in the soft cheese production as the detection of such genotype in the final product, and not in the raw material, suggests that the dairy cows were not contaminated. We have no evidence of possible contact points between the two Italian food producers that could explain the presence of such plasmid-carrying genotypes in both facilities. However, given the restricted geographical area in which the facilities are located, the variety of sources (e.g., effluents and other residues^36^) by which pathogens may enter the food-producing environments, and the ubiquitous nature of *Klebsiella* species^34^, we could imagine that the environment may have played a role as a vehicle for spread of *K. pneumoniae* ST3254 across both food settings.

Besides emerging insights on the role of *Klebsiella* spp. as a vehicle for the dissemination of virulence genes along the food chain, our strains reported low AMR burden. A susceptible phenotype was noticed for almost all isolates, reporting susceptibly to all tested antibiotics except ampicillin, for which resistance is an intrinsic feature of *Klebsiella* spp. due to the presence of chromosomally encoded beta-lactamases^12,25,57,58^. Similar resistance rate of ampicillin is consistent with previous reports on food samples worldwide distributed from various sources^28,59–61^. Similarly, scarce therapeutical concern should arise from low resistance rates of azithromycin (one *K. oxytoca* and one *K. grimontii*), which is traditionally underused as standard treatment option for enterobacterial infections^62^.

Our study also highlighted a high variability at strain level across the *Klebsiella* spp. population as reflected by the extensive range of STs found, half of which were novel, probably due to the scarcity of studies focused on food productions. This represents a first step towards a deeper understanding of relevant STs colonizing those hitherto unexplored food niches. The public genome collection hosted in the PathogenWatch platform (https://pathogen.watch/) offered a first glance to explore and contextualize our known and novel STs at local, national, or global levels. Many *K. pneumoniae* STs have been occasionally isolated from human clinical isolates, with major STs detected (ST4242 and ST3254) spotted in Norway and US, and ST107, a globally distributed ST, also reported in Italy. Likewise, *K. oxytoca* ST36 and ST37 have been found in human samples from Europe, whereas *K. michiganensis* ST381 from UK and ST50/ST210 from US, and *K. pasteurii* ST311 from Nigeria.

Analyzing local data within a broader ecological context is essential to understand the global circulation of genotypes across different niches or hosts. The cgMLST typing approach allowed us to compare local strains in the context of hundreds of public *Klebsiella* spp. sequences from other sources. We observed that high core genome proximity may exist between human and food strains of *K. pneumoniae* or *K. oxytoca* (~ 1.5% of variant differences). This suggests that in addition to the human-animal transmission of *Kp* strains^34,52^, transmission may also occur from food to humans; however, more sampling and prospective designs will be needed to define the directionality of transmission.

In conclusion, this study contributed to extend the body of knowledge on the prevalence and features of concern of *Klebsiella* spp. strains in the food chain.

Following a one health approach, we provide evidence of *Klebsiella* presence and circulation of closely related genotypes and plasmid-encoded genes between interconnected settings (farm, food, and human). The carriage of AMR and virulence traits by *Klebsiella* along the food chain should not be underestimated, as such features could be transferred to other bacteria in the same niche (e.g., human gut or environment) and help them colonize the gut of individuals who might develop invasive diseases. More active surveillance of *Klebsiella* in the food chain would help to gain more knowledge on *Klebsiella*, especially *K. pneumoniae*, as a foodborne pathogen.

## Methods

### Sampling procedure

In this study, 1,170 samples were collected between January 2020 and May 2021 from six batches of dairy and pork meat-based productions in two artisanal RTE small-scale factories both located in the region of Emilia Romagna (Northern Italy). The dairy production corresponded to a soft cheese prepared with pasteurized cow milk^63^. The other production was represented by a fermented dried sausage, so-called “salami”, prepared using pork meat from an Italian swine autochthonous breed with no addition of starter cultures or nitrites/nitrates. Within both artisanal facilities, the six commercial batches were produced in different months, to reflect a whole seasonality cycle, but respecting the same traditional methods from raw materials to the end of the production line (cheese and salami end-products). The sampling started at the artisanal facilities from raw materials to final products collected over each key processing stage, plus environmental swabs picked up from surfaces, machines, and operator’s gloves, while the food was managed within the processing rooms (Supplementary Fig. S3). The latter were collected before cleaning and disinfection procedures, by swabbing a 100 cm2 area with a sterile cotton swab (Copan Italia, Brescia, Italy) that had been moistened in 10 mL of saline solution (0.9% NaCl). A total of 420 and 750 samples were collected from salami and cheese productions, respectively (Table 1).

**Table 1:** Overview of the 1170 samples collected across six batches of salami and cheese production in the artisanal facilities.

Artisanal RTE food production	Sample origin	Description	Number (n)
Salami	Environmental	Wall inside stuffing, drying and ripening area	90
		Manhole inside stuffing, drying and ripening area	90
		Processing surfaces inside stuffing area	30
		Filler stuffer machine located within the stuffing area	30
	Raw materials	Minced meat mixture managed within the stuffing area	30
		Salami stored for 1 week inside drying area	30
		Salami stored for 3 weeks inside ripening area	30
		Salami stored for 10 weeks inside ripening area	30
		Salami stored for 18 weeks inside ripening area	30
	Final product	Ripened salami (stored for 28 weeks inside ripening area)	30
Total			420
Cheese	Environmental	Wall inside warm, maturation and packaging area	90
		Manhole inside warm, maturation and packaging area	90
		Gloves of workers inside the packaging area	30
	Raw materials	Raw and pasteurized milk, calf rennet and raw cheese stored within warm and maturation room	150
	Final product	Cheese packed on the same day (“day 0”) of production along with stored cheese at day 4, 8, 11 and 15 at 2 °C, 8 °C and 2 °C for 5 days followed by 8 °C for 10 days	390
Total			750

### Isolation of *Klebsiella* spp

For both cheese and meat productions, one aliquot of 25 g from samples of raw materials as well as intermediate and final products along with swabs picked up from the processing environment were enriched in buffered peptone water (Thermo Scientific™) at 37 °C overnight. A 10-μL loopful of the overnight-enriched culture was streaked onto MacConkey agar (Thermo Scientific™) and incubated again at 37 °C for 24 h. Colonies showing the typical pink mucoid morphology related to *Klebsiella* spp. were picked and submitted to biochemical test (RapID™ ONE System, Thermo Scientific™) for preliminary species confirmation. DNA extraction (Chelex method^64^) was then performed on strains identified as *K. pneumoniae* and *K. oxytoca* for species identification by multiplex polymerase chain reaction^31^ (multiplex-PCR). *Klebsiella* spp. positive isolates (n = 75) were stored at − 80 °C in Brain Heart Infusion broth (BHI, Thermo Scientific™) with 20% glycerol until further use.

### Antimicrobial susceptibility testing

After species confirmation, antibiotic susceptibility testing of *Klebsiella* spp. strains was carried out using the Sensititre™ EUVSEC ready to use plates (Thermo Scientific, USA) and following the gold standard broth microdilution phenotypic assay^65^. Following the manufacturer’s protocol, isolates were tested against 13 antibiotics: trimethoprim (TMP, from 0.25 to 32 mg/L), ciprofloxacin (CIP, from 0.015 to 8 mg/L), tetracycline (TET, from 2 to 64 mg/L), meropenem (MERO, from 0.03 to 16 mg/L), azithromycin (AZI, from 2 to 64 mg/L), nalidixic acid (NAL, from 4 to 128 mg/L), cefotaxime (FOT, from 0.25 to 4 mg/L), chloramphenicol (CHL, from 8 to 128 mg/L), tigecycline (TGC, from 0.25 to 8 mg/L), ceftazidime (TAZ, from 0.5 to 8 mg/L), colistin (COL, from 1 to 16 mg/L), ampicillin (AMP, from 1 to 64 mg/L) and gentamicin (GEN, from 0.5 to 32 mg/L). The isolates were defined as susceptible or resistant according to the clinical breakpoints (CBP) established by the European Committee on Antimicrobial Susceptibility Testing (EUCAST), except for those antimicrobials for which a CBP was not available^32^. In particular, CBPs of the Clinical and Laboratory Standard Institute (CLSI) were applied for tetracycline, azithromycin and nalidixic acid^66^.

### DNA extraction and whole genome sequencing (WGS)

*Klebsiella* spp. isolates confirmed by biochemical test and PCR were grown overnight at 37 °C on BHI broth (Thermo Scientific™). DNA extraction for WGS purposes was performed with the MagAttract HMW DNA Kit (Qiagen, Hilden, Germany) according to manufacturer’s instructions. BioSpectrometer fluorescence (Eppendorf) was used to measure the purified DNA concentration and the quality parameter ratio 260/280. Libraries were prepared using the Nextera XT DNA Library Preparation Kit (Illumina, Milan, Italy) and the whole genome of selected isolates was paired end sequenced (250 bp) using the MiSeq platform (Illumina). Reads quality assessment was carried out with Kraken2 v2.1.1 (https://github.com/DerrickWood/kraken2). Raw reads with good quality were pre-processed and de novo assembled using fq2dna v21.06 (https://gitlab.pasteur.fr/GIPhy/fq2dna), which also performed the post-processing scaffold sequence accuracy assessment. Contiguity metrics was plotted with ggplot2 package in R v4.1.2 (https://cran.r-project.org/).

### Genome-based taxonomic assignment and sequence typing

The taxonomy at species levels, previously assigned with biochemical and molecular testing, was then assessed by WGS using different tools: Kraken2 v2.1.1 (with the pre-buit MiniKraken DB) (https://github.com/DerrickWood/kraken2) for taxonomic classification of reads, ReferenceSeeker v1.7.3 for average nucleotide identity (ANI), based search of closely related reference genomes from RefSeq (https://www.ncbi.nlm.nih.gov/refseq), and Kleborate v2.0.0 (https://github.com/katholt/Kleborate) for rapid and accurate species and subspecies prediction based on Mash distances calculated against a taxonomically curated genome set of *Klebsiella* spp. as well as other Enterobacterales assemblies. Results from the three different tools were combined to provide a more accurate species assignment. For genomes with discordant results (n = 7), hits from Kleborate were retained due to the high specificity of this tool for species and subspecies identification for *K. pneumoniae* and its associated species complexes.

Known sequence types (STs) were assigned using Kleborate and MLST v2.19.0 (https://github.com/tseemann/mlst) based on the multilocus sequence typing (MLST) schemes hosted in the BIGSdb-Pasteur (https://bigsdb.pasteur.fr/klebsiella/) and pubMLST^67^. *K. pneumoniae* and *K. oxytoca* isolates with unknown alleles and sequence types were submitted to BIGSdb-Pasteur.fr and pubMLST.org platforms, respectively, for the definition of novel alleles and STs.

### Antimicrobial resistance and virulence genes detection, and location in the genomic context

The software Kleborate was used to screen the *Klebsiella* spp. genome assemblies against a curated database of key virulence and AMR loci using a minimum threshold of 90% for nucleotide identity and gene coverage^68^. New variants of the aerobactin and yersiniabactin sequence types found in *K. pneumoniae* isolates were submitted to BIGSdb-Pasteur.fr for definition of the novel AbST and YbST. Plasmid sequences were reconstructed from genome assemblies using the MOB-recon tool of MOB-suite v3.0.1 (https://github.com/phac-nml/mob-suite)^69^. The typing option was also enabled to predict plasmids’ mobility. Moreover, a plasmid multilocus sequence typing (pMLST) was performed on the subset of *K. pneumoniae* draft genomes using the pMLST Web tool (http://cge.cbs.dtu.dk/services/pMLST/) selecting the IncF RST configuration.

### Comparison of virulence-related plasmids with public data

To draw a broader picture of the possible ecological niches in which virulence-related plasmids similar to those herein detected can circulate, a pairwise comparison of plasmids from this study and selected sequences from public databases (e.g., Microreact and NCBI) was performed with fastANI v1.33 (https://github.com/ParBLiSS/FastANI). The sequences of virulence-related plasmids were also annotated with PROKKA v1.14.5 (https://github.com/tseemann/prokka)^70^ and a manual curation of hypothetical proteins was performed searching for homologous protein in the NCBI database (https://blast.ncbi.nlm.nih.gov/Blast.cgi). Annotated proteins were then submitted to Clinker v0.0.23 (https://github.com/gamcil/clinker) for interactive visualization of protein clusters and alignments.

### Pan-genome calculation

The entire gene repertoire of *Klebsiella* strains from the artisanal productions was assessed reconstructing the pangenome with Panaroo v1.2.3 (https://github.com/gtonkinhill/panaroo), selecting the sensitive mode with 95% of sequence identity and core-gene thresholds^71^. The resulted alignment was further used to build a Maximum-likelihood (ML) phylogenetic tree with IQ-Tree using the substitution model GTR + F + R8 in v2.0.6 (https://github.com/Cibiv/IQ-TREE)^72,73^. The distribution of core and accessory genes was observed across the phylogenomic tree and the samples’ metadata on the Phandango website (https://jameshadfield.github.io/phandango/#/) for interactive pangenome visualization.

### Gene-by-gene approach and phylogenetic analyses

To provide insight into strains relatedness, the ML tree inferred on core genome multiple sequence alignments was visualized with ITOL (https://itol.embl.de/) along with samples metadata (e.g., food product, batch, and sample origin), STs and virulence information. For KplaSC, as no MLST schemes has been defined yet, arbitrary identifiers (from new1 to new6) were attributed to the different strains based on the core gene phylogeny branches (Fig. 2).

A core genome MLST (cgMLST) approach was then used to assess the genetic distance of genotypes from the artisanal production within an extended context of public strains selected from the National Centre for Biotechnology Information (NCBI, https://www.ncbi.nlm.nih.gov/) and BIGSdb-Pasteur (https://bigsdb.pasteur.fr/klebsiella). The *K. pneumoniae* strains were uploaded on the BIGSdb-*Kp* database and compared with the *K. pneumoniae* strains belonging to the same ST (n = 15) using the 629 loci cgMLST scheme, scgMLST629_S, hosted and curated on the platform^33^. There was no cgMLST scheme available to compare KoSC and KplaSC genotypes. We therefore used the chewBBACA suite v2.8.5 (https://github.com/B-UMMI/chewBBACA) and built dedicated cgMLST schemes to separately analyze KoSC and KplaSC strains. For scheme creation, all public genomes were firstly downloaded from the NCBI genome repository, representing the overall population of KplaSC (*K. planticola* and *K. ornithynolitica*) and KoSC (*K. oxytoca*, *K. michiganensis*, *K. grimontii* and *K. pasteurii*) isolated worldwide. Further, eight strains from the Collection of Institut Pasteur (CIP, Paris, France) (n = 1 *K. planticola*, n = 2 *K. ornithynolitica* for KplaSC; n = 1 *K. oxytoca*, n = 1 *K. michiganensis*, n = 2 *K. grimontii* and n = 1 *K. pasteurii* for KoSC), and six *K. oxytoca* reference strains (ATCC® 700,324™, ATCC® 13,182™, ATCC® 49,131™, ATCC® 51,983™, ATCC® 13,030™ and ATCC® BAA-3059™; retrieved at https://www.atcc.org/) were included in the public dataset. A first screening of public genomes was carried out based on assemblies’ quality metrics, by filtering out sequences with more than 500 contigs, total genome size outside the typical length of *Klebsiella* spp. (< 4.5 Mb and > 6.5 Mb) and N50 lower than 30,000. Strains with mixed species as calculated by the ribosomal MLST based species identification tool (https://pubmlst.org/species-id) were also excluded^74^. The remaining assemblies were then clustered together with artisanal food genomes based on pairwise MASH-based distance (https://gitlab.pasteur.fr/GIPhy/JolyTree), to carefully select reference genomes for the scheme construction. Representative genomes for each cluster were selected based on best contiguity metrics, and the diversity of available metadata (location and collection date) and of sequence types (classical MLST or rMLST where MLST was not available). This selection was aimed at embracing the broadest population diversity while preventing redundancy of the dataset. The selected references, 51 and 104 genomes for KplaSC and KoSC, respectively, were used to build a cgMLST scheme for each species complex (see Supplementary Table S11 for public genomes list and metadata from NCBI, downloaded with NCBImeta v0.8.3, https://github.com/ktmeaton/NCBImeta). The cgMLST schemes were built following recommendations and steps available in the chewBBACA guidelines (https://github.com/B-UMMI/chewBBACA#i-whole-genome-multilocus-sequence-typing-wgmlst-schema-creation). Paralogous loci were removed from the scheme and the loci present in at least 99% of samples were extracted. The quality of core loci was estimated with the chewBBACA *Schema Evaluation* module and the reported loci with high allele variability were excluded from the scheme. The cgMLST schemes were finally composed of 3,272 loci for KoSC and 2,957 loci for KplaSC. The schemes were deposited in a Zenodo repository for public access (10.5281/zenodo.7477602). We used these schemes to define the allelic profiles of strains from food production facilities and a collection of public strains from other sources (Supplementary Table S11) and with the lowest genetic distance based on MASH analysis (https://gitlab.pasteur.fr/GIPhy/JolyTree). Pairwise distances between allele profiles were then analyzed using the Minimum Spanning Tree (MST)-based clustering tool MSTclust v0.21b (https://gitlab.pasteur.fr/GIPhy/MSTclust) to identify clusters of strains that share a closely related genotype (e.g., ~ 1.5% of allele distances across the dedicated cgMLST scheme).

## Supplementary Information

Supplementary Tables.

Supplementary Figures.

## Data Availability

The paired-end reads included in this study have been deposited in the European Nucleotide Archive (ENA) at EMBL-EBI under accession number PRJEB56668 (https://www.ebi.ac.uk/ena/browser/view/PRJEB56668). The KoSC and KplaSC schemes were deposited in a Zenodo repository for public access (10.5281/zenodo.7477602).
