# Supplementary material for: Genomic features of Klebsiella isolates from artisanal ready-to-eat food production facilities
Source: Sci Rep. 2023 Jul 6;13:10957. doi: 10.1038/s41598-023-37821-7 (PMC10326032; doi:10.1038/s41598-023-37821-7)
Supplement: Supplementary file 2 — Supplementary Figures. [file 41598_2023_37821_MOESM2_ESM.pdf]

Manuscript title: Genomic features of *Klebsiella* isolates from artisanal ready-to-eat food production facilities

Authors: Cecilia Crippa, Frédérique Pasquali, Carla Rodrigues, Alessandra De Cesare, Alex Lucchi, Lucia Gambi, Gerardo Manfreda, Sylvain Brisse and Federica Palma

Supplementary Figure S1: Quality metrics boxplots of 73 *Klebsiella* spp. strains.

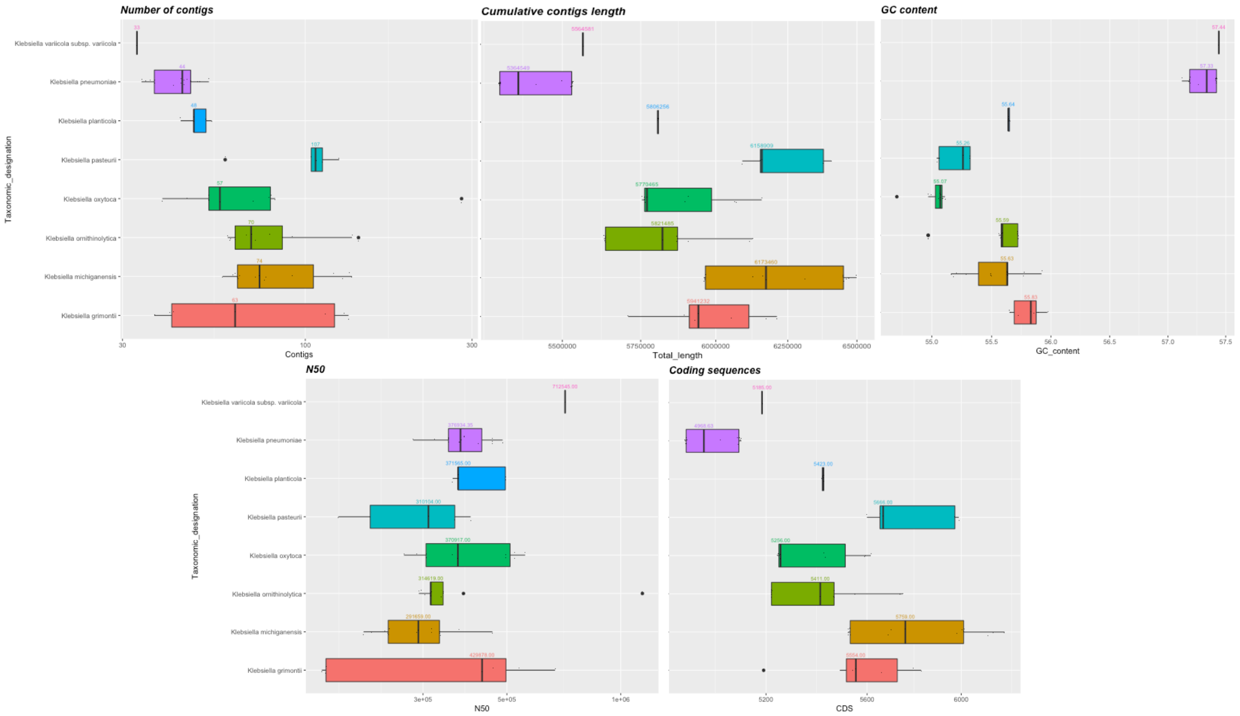

Boxplots of assembly (number of contigs, cumulative contigs length, GC content and N50) and annotation (coding sequences) quality metrics for *Klebsiella* spp. strains belonging to each species complex. The boxes display the median (50%) as well as the first (25%, Q1) and the third (75%, Q3) quantile. Values that are either less than Q1 – 1,5 * IQR or greater than Q3 + 1,5 * IQR are considered outliers.

Supplementary Figure S2: Pangenome visualization of 73 *Klebsiella* spp. strains.

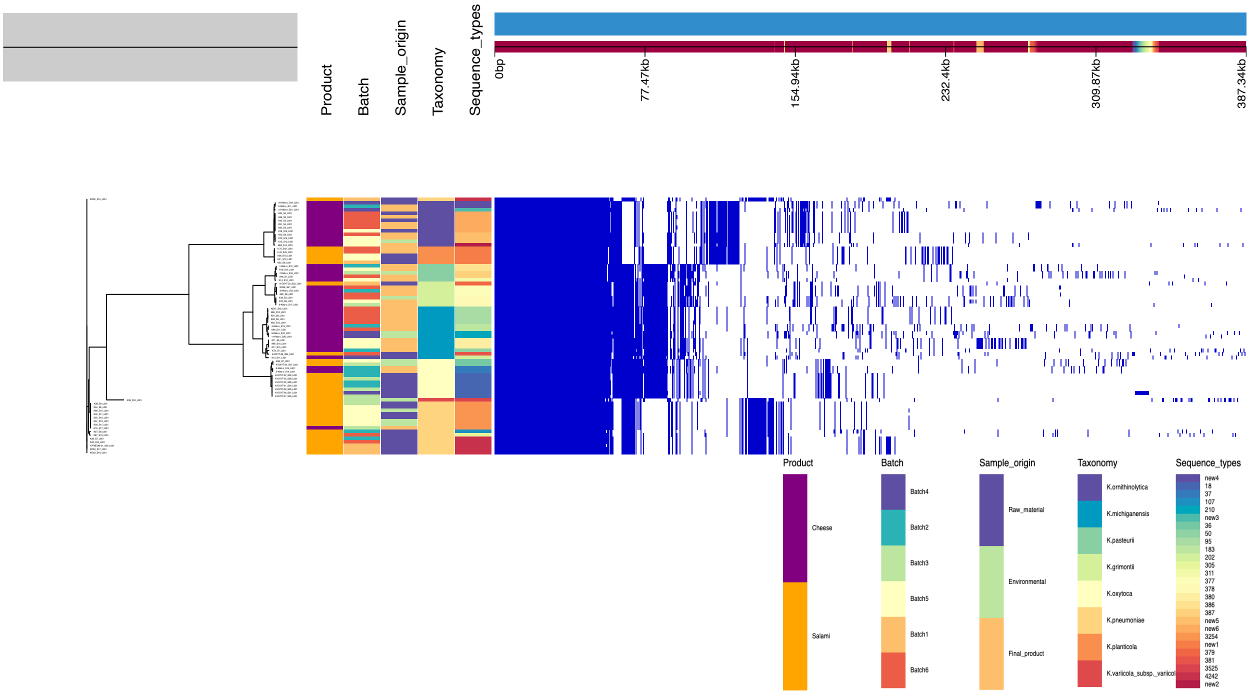

Interactive pangenome visualization of 73 *Klebsiella* spp. strains obtained by Phandango website. A core-genes phylogenetic tree is inferred with strains associated metadata (food product, batch, sample origin, taxonomy and STs) as well as pangenome content (core and accessory genes).

Supplementary Figure S3: **Process flowcharts of cheese and salami artisanal food productions.**

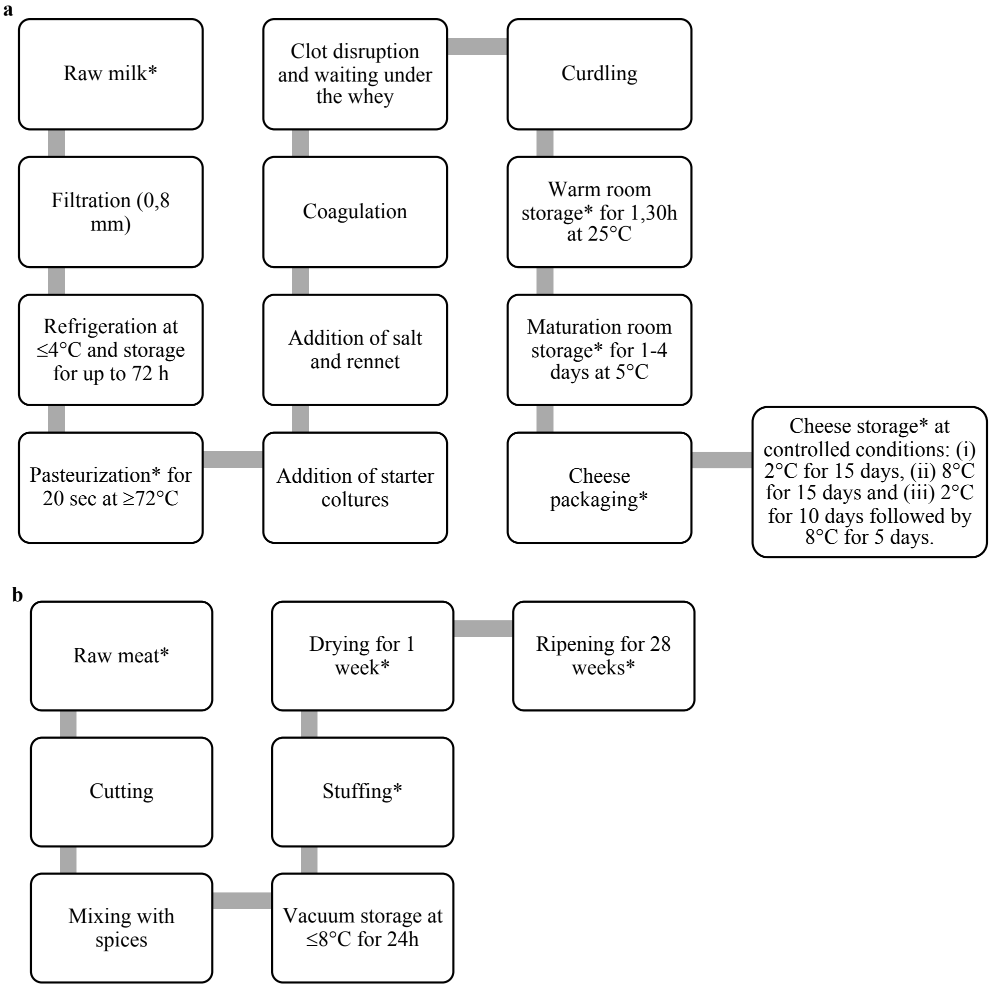

Flowcharts summarizing key processing stages in cheese (a) and salami (b) productions realized in the artisanal plants. Processing areas and stages where samples from food (raw materials, intermediate and/or final product) and processing environment were taken have been marked by adding an asterisk within the boxes.
